# Landscape of gut microbiota and metabolites and their interaction in comorbid heart failure and depressive symptoms: a random forest analysis study

**DOI:** 10.1128/msystems.00515-23

**Published:** 2023-10-26

**Authors:** Kai Huang, Jiahao Duan, Ruting Wang, Hangfeng Ying, Qinwen Feng, Bin Zhu, Chun Yang, Ling Yang

**Affiliations:** 1 Department of Cardiology, The Third Affiliated Hospital of Soochow University, Changzhou, China; 2 Department of Critical Care Medicine, The Third Affiliated Hospital of Soochow University, Changzhou, China; 3 Department of Anesthesiology and Perioperative Medicine, The First Affiliated Hospital of Nanjing Medical University, Nanjing, China; Istanbul Medipol University School of Medicine, Istanbul, Turkey

**Keywords:** heart failure, depression, gut microbiota, gut metabolites, random forest

## Abstract

Depression is an individual risk factor for poor prognosis in patients with heart failure (HF). Recent studies show that gut microbiota and metabolites play a critical role in comorbid HF and depressive symptoms. We recruited 95 subjects including 35 HF patients with depressive symptoms (HF-DS), 36 HF patients without depressive symptoms (HF-NDS), and 24 healthy controls (HC). The 16S rRNA, metagenome sequencing, and untargeted metabolomic analysis were employed to test fecal samples. Our analysis found a significant difference in composition of gut microbiota in HF-DS, HF-NDS, and HC populations. At the genus level, *Mediterranea*, *Tolumona*, and *Parabacteroides* were significantly increased in HF-DS patients compared with HF-NDS patients, while *Pedobacter*, *Azospirillum*, and *Ruminiclostridium* were significantly decreased. Furthermore, anti-inflammatory mediators (abietic acid, quinic acid, linoleic acid, etc.) and neurotransmitters (catechin, serotonin, tryptamine, phenylethylamine, etc.) were reduced in HF-DS. The enrichment analysis revealed that the gut microbiota highly conformed to the functional pathways of metabolites, and amino acid-related metabolism, fatty acid-related metabolism, and cAMP signaling pathways may be crucial biological mechanisms involved in the development of comorbid depression and HF. Finally, *Cloacibacillus* and alpha-tocopherol were determined as diagnostic markers for HF-DS patients.

**IMPORTANCE:**

There is increasing evidence that alterations in gut microbial composition and function are associated with cardiovascular or psychiatric disease. Therefore, it is meaningful to investigate the taxonomic and functional characterization of the microbiota in HF patients who also have depressive symptoms. In this cross-sectional study, *Cloacibacillus* and alpha-tocopherol were determined as new diagnostic markers. Furthermore, intestinal microecosystem disorders are closely linked to depressive symptoms in HF patients, providing a new reference viewpoint for understanding the gut-heart/brain axis.

## INTRODUCTION

Depression is a prominent and debilitating concomitant symptom that affects patients with heart failure (HF), significantly linked to reduced quality of life and poor clinical prognosis (1). Many risk factors, including gender, living alone, alcohol abuse, platelet response activation, inflammation, and neuroendocrine dysregulation, may be associated with HF comorbid depression (2). In addition, our prior studies indicated that vagus nerve, gut microbiota, etc. may have a negative overlapping impact on depressive symptoms in HF patients (3, 4). These complex unwanted factors increase the clinical heterogeneity of conditions and raise challenges in diagnosing and treating HF patients with depression.

Gut microbiota is the community of microorganisms that reside in the intestine, which plays a crucial role in sustaining bodily health by participating in metabolic networks and regulating the immune system (5). Currently, several gut theories postulate that gut microbiota and metabolites are involved in the development of cardiovascular or psychiatric diseases via the gut-heart or gut-brain axis (6, 7). Although there is no ultimate conclusion, the structure of the microbiota is significantly modified in patients with HF or depression when compared to the healthy population. In HF patients, the levels of pathogenic bacteria such as *Campylobacter*, *Shigella*, *Salmonella*, and *Yersinia enterocolitica* are increased, while beneficial bacteria such as *Bifidobacterium* and *Lactobacillus* are reduced (8). Furthermore, HF patients have lesser bacteria that produce butyrate, which has a local anti-inflammatory effect in the intestinal mucosa (9). Interestingly, lower fecal butyrate and reduced counts of *Roseburia*, *Romboutsia*, and *Prevotella* are linked to depressive symptoms (10). Furthermore, fecal transplantation experiments have suggested that transplanting depressed microbiota into germ-free mice can induce depressive-like behavior, which explains the causal role of gut microbiota in the pathogenesis of depression (11, 12). Therefore, revealing new mechanisms of HF comorbid with depression by determining the intestinal environment is a viable strategy.

Most recently, 16S rRNA sequencing has been reliably applied to evaluate community differences, species composition, and diversity analysis (13). However, metagenome sequencing can provide more in-depth taxonomic characterization (14). In this research, we conducted 16S rRNA and metagenome sequencing of fecal samples from HF patients with depressive symptoms (HF-DS), HF patients without depressive symptoms (HF-NDS), and healthy control (HC) to distinguish the signatures of gut microbiota and their functional potential. More importantly, the majority of microbial-host interactions are achieved by metabolic signals, and changes in metabolites show changes in colony function under specific conditions, helping to clarify the complex mechanism and causality between “microbial-host” (15). We therefore conducted a gas chromatography-mass spectrometry (GC-MS)-based metabolomic analysis of feces. Enrichment analysis and association analysis of species and metabolites were conducted to reveal the potential gut microbiota and their functional metabolites linked to depressive symptoms after HF. Finally, combined biomarkers to differentiate HF-DS and HF-NDS were determined based on a random forest algorithm.

## MATERIALS AND METHODS

### Study cohorts

In this cross-sectional study, we enrolled 96 patients with HF who attended the Department of Cardiovascular Medicine at the Third Affiliated Hospital of Soochow University between December 2020 and November 2021, and 24 HC who had a physical examination. HF patients met the 2016 ESC guideline criteria for the diagnosis of heart failure (16), an integration of at least one of the following: elevated natriuretic peptide levels; objective evidence of cardiogenic pulmonary or body circulation stasis, including imaging (e.g., chest radiograph and echocardiogram); and resting or stress hemodynamic monitoring (e.g., right heart catheter and pulmonary artery catheter). Furthermore, the following participants were excluded from the study: (i) taking probiotics or antibiotics within 2 weeks before the trial; (ii) comorbid with autoimmune diseases, infections, malignancies, and acute coronary syndromes; (iii) comorbid with cerebrovascular embolism, respiratory diseases, and other diseases influencing the intestinal microbiota; and (iv) unable to complete depression scale evaluations or to retain feces. HF patients were scored by the PHQ-9 self-rating scale, a nine-item questionnaire that is often used to screen patients for depressive symptoms. Ultimately, 71 HF patients were recruited in this research, with scores ≥10 in the HF-DS group (*n* = 35) and <10 in the HF-NDS group (*n* = 36). All patients were treated based on the current HF management guidelines, and important clinical information was obtained from electronic medical records. This study was authorized by the Ethics Committee of the Third Affiliated Hospital of Soochow University (No. 2021140) and complied with the principles outlined in the Declaration of Helsinki, and all participants (or their immediate family members) gave informed consent.

### Sample preparation

We obtained fresh fecal samples (5–10 g) from all subjects within 48 h of admission and immediately transferred to collection tubes fitted with fecal stabilizers and stored in a −80°C cryogenic refrigerator until subsequent testing.

### 16S rRNA sequencing and analysis

Fecal genomic DNA was extracted with a DNA extraction kit (Magen, Guangdong, China), and DNA concentration and integrity were determined by NanoDrop 2000 spectrophotometer (Thermo Fisher Scientific, Waltham, MA, USA) and agarose gel electrophoresis, respectively. Subsequently, PCR amplification of the V3-V4 high variant region of the bacterial gene was conducted in a 25-µL reaction system using specific primers with barcode (343F: 5′-TACGGRAGGCAGCAG-3′; 798R: 5′-AGGGTATCTAATCCT-3′) according to the selection of the sequenced region (17). PCR products were purified with Agencourt AMPure XP beads (Beckman Coulter Co., USA) and quantified with the Qubit dsDNA Detection Kit (Life Technologies, Cat. Q32854). End-pairing cycle sequencing was carried out on Illumina NovaSeq6000 (Illumina Inc., San Diego, CA, USA) at 250 bases per cycle after concentration adjustment (18).

The raw data were analyzed as follows (19): (i) the primer sequences were cut by Cutadapt software; (ii) the qualified double-ended raw data from the previous step were then subjected to quality filtering, noise reduction, splicing, and chimera removal according to the default parameters of QIIME2 using DADA2 to acquire representative sequences and ASV abundance tables; (iii) the representative sequences of each ASV were chosen using the QIIME2 package, and all representative sequences were evaluated with the Silva (version 138) database for annotation.

### Metagenome sequencing and analysis

The metagenome sequencing and data analysis were performed by OE Biotech Co., Ltd. (Shanghai, China). Macrogenomic libraries were constructed using a TruSeq DNA sample preparation kit (Illumina, San Diego, CA, USA), and paired-end sequencing was conducted for each sample on Illumina NovaSeq6000 (Illumina Inc., San Diego, CA, USA) (20). The sequencing primers were subjected to cluster generation, template hybridization, isothermal amplification, linearization, blocking denaturation, and hybridization per the workflow stipulated by the service provider.

The raw data were analyzed as follows (21–23): (i) Trimmomatic (v0.36) excised junctions and filtered out low-quality bases and removed reads containing ambiguous bases; (ii) double-ended reads were evaluated with the host genome using bowtie2 (v2.2.9) for quality control of de-hosting contamination; (iii) macrogenome splicing assembly by MEGAHIT (v1.1.2) and ORF prediction by prodigal (v2.6.3) for contigs higher than or equal to 200 bp or 500 bp; (iv) construction of non-redundant gene sets using CDHIT (v4.5.7) for predicted genes in all samples with clustering parameters of 95% identity between sequences and 90% coverage; and (v) using bowtie2 (v2.2.9), the clean reads of each sample were evaluated with the non-redundant gene set separately (95% identity), and the abundance information of the genes in the corresponding samples was counted.

### Untargeted metabolomic profiling

The frozen feces was blended with methanol (Thermo Fisher Scientific, Waltham, MA, USA) and extracted by sonication in an ice water bath for 20 min. The extracts were centrifuged at 4°C (13,000 rpm) for 10 min and the supernatant was dried. The extracts were then mashed with methoxyamine hydrochloride pyridine (CNW Technologies GmbH, Düsseldorf, Germany) solution and derivatized. The derivatized samples were evaluated on an Agilent 7890B GC system coupled to an Agilent 5977A MSD system (Agilent Technologies Inc., CA, USA). The DB-5MS-fused silica capillary column (30 m × 0.25 mM × 0.25 µM, Agilent J & W Scientific, Folsom, CA, USA) was used to isolate the derivatives with a constant gas flow rate of 1 mL/min. Mass spectrometry data were obtained in full scan mode (m/z 50–500), and the samples were injected into the mass spectrometer at regular intervals throughout the examination. The samples were infused into the mass spectrometer conventionally during the analysis to offer a set of data that could be assessed for reproducibility. The metabolic characterization of the data was subsequently analyzed per the LUG database. After screening each sample according to an internal criterion of RSD > 0.3, all peak signal intensities were divided and normalized.

### Functional investigation of microbiota and metabolites

The KEGG database was constructed to comprehend the functions and interactions of genes, proteins, and metabolites in biological systems (e.g., cells and tissues). In this research, we annotated the representative sequences of gene sets with the KEGG database by DIAMOND (v0.9.7), and BLAST comparison parameters were set with an expectation of E < 1e^−5^ to acquire the functional abundance at each level. For metabolites, enrichment analysis was conducted using their KEGG IDs to obtain metabolic pathway enrichment results. Hypergeometric tests were used to determine pathway entries that were substantially enriched in significantly differentially expressed metabolites in comparison to the entire background.

### Statistical analysis

Differences in clinical characteristics between groups were identified using Student’s *t*-test or Kruskal–Wallis test. Microbial characteristics (species and metabolites) that appeared in less than 20% of the samples were excluded before statistical examination of the relative abundance data. The Wilcoxon rank sum test was used to assess the characteristics (α diversity and β diversity) and abundance of gut microbes in each group. The differential metabolites screen criteria were variable important in projection (VIP) value >1 for the first principal component of the orthogonal partial least squares-discriminant analysis (OPLS-DA) model and *P* < 0.05 for the *t*-test. Associations between gut microbiota and metabolites were evaluated by Spearman’s rank correlation and corrected for significance using the Benjamini-Hochberg procedure. Groups were evaluated for statistically significant species and functional differences by linear discriminant analysis effect size (LEfSe), which used the nonparametric factorial Kruskal–Wallis test, Wilcoxon rank sum test, and LDA to determine the biological and functional markers that were enriched for differences between multiple metadata categories. To identify a set of gut microbiota and metabolites used to distinguish depressive symptoms in HF patients, a random forest model was used to individually rank each type of profile and verified by a 10-fold stratified cross-validation test. The optimal number of discriminative markers for each profile was calculated using a recursive feature elimination method with default parameters using five different random seeds. Receiver operating characteristic (ROC) curves constructed using fivefold cross-validation were employed to test the performance of combined markers for enteric bacteria and metabolites. The above analyses were statistically and graphically conducted by R (v4.1.3) and SPSS (v19.0). Unless otherwise stated, all statistical tests are two-tailed, and *P* < 0.05 was deemed statistically significant.

## RESULTS

### Study design and population characteristics

To assess the link between gut microecological disorders and depressive symptoms in HF patients, we obtained fecal samples from 120 subjects. Per the inclusion and exclusion criteria, 95 subjects, including HF-DS (*n* = 35), HF-NDS (*n* = 36), and HC (*n* = 24), were finally recruited for 16S rRNA sequencing, metagenome sequencing, and untargeted metabolomic analysis. The baseline characteristics of the participants are presented in Table 1, and no statistically significant differences in smoking, alcohol consumption, and body mass index were observed among the three groups. Similar to previous reports, women were more likely to have depressive symptoms in HF patients (24). Regarding cardiac function, N-terminal brain natriuretic peptide precursor levels were significantly higher in HF-DS patients than HF-NDS patients, while there were no significant differences in left ventricular ejection fractions and heart rate. Compared with HC group, alanine aminotransferase, aspartate transaminase (AST), lactate dehydrogenase, creatinine, and urea were high in HF patients. Interestingly, there was no significant difference between HF-DS and HF-NDS in hypertension, coronary artery disease, and atrial fibrillations.

**TABLE 1:** Baseline characteristics of the study population^*a*^

	HF-DS (*n* = 35)	HF-NDS (*n* = 36)	HC (*n* = 24)	*P*
Age (years)	69.37 ± 10.12	65.56 ± 11.75	57.08 ± 9.34	<0.001^bc^
BMI (kg/m^2^)	24.17 ± 2.67	25.45 ± 4.18	24.24 ± 2.32	0.410
Male (%)	16 (45.71%)	27 (75.00%)	12 (50.00%)	<0.05^ac^
Smoke (%)	10 (28.57%)	12 (33.33%)	4 (16.67%)	0.358
Drinking (%)	8 (22.86%)	9 (25.00%)	1 (4.17%)	0.099
PHQ-9	12.03 ± 2.02	5.50 ± 1.66	–^*b*^	<0.001^a^
Hypertension (%)	21 (60.00%)	24 (66.67%)	11 (45.83%)	0.271
CAD (%)	16 (45.71%)	12 (33.33%)	–	0.337
AF (%)	16 (45.71%)	13 (36.11%)	–	0.474
NYHA class				0.015^a^
II class	4 (11.43%)	13 (36.11%)	–	
III class	24 (68.57%)	13 (36.11%)	–	
IV class	7 (20.00%)	10 (27.78%)	–	
LVEF(%）	44.43 ± 14.75	45.17 ± 13.92	63.42 ± 2.24	<0.001^bc^
NT-proBNP (pg/mL)	2960.00 (1110.00, 8120.00)	1615.00 (893.00, 3535.00)	–	0.022^a^
HR (bpm)	86.46 ± 18.82	87.50 ± 24.51	71.25 ± 12.97	<0.001^bc^
ALT (U/L)	20.70 (12.20, 41.70)	22.3 0 (16.03, 32.68)	15.65 (13.00, 21.88)	0.066
AST (U/L)	25.80 (18.10, 35.50)	21.30 (17.35, 32.25)	19.20 (16.45, 22.18)	<0.05^b^
LDH (U/L)	198.00 (173.00, 243.00)	199.50 (171.25, 232.50)	158 (141.50, 185.75)	<0.001^bc^
STB (μm/L)	16.30 (11.30, 21.90)	13.95 (10.55, 20.93)	11.65 (10.03, 14.78)	<0.05^b^
TC (mmol/L)	3.80 ± 0.89	4.39 ± 1.09	4.74 ± 0.72	<0.001^ab^
TG (mmol/L)	1.11 ± 0.43	1.42 ± 0.56	1.50 ± 0.67	<0.05^ab^
HDL (mmol/L)	1.05 ± 0.27	1.07 ± 0.25	1.27 ± 0.30	<0.05^bc^
LDL (mmol/L)	2.09 ± 0.73	2.53 ± 0.82	2.63 ± 0.49	<0.01^ab^
Urea (mmol/L)	6.62 (5.08, 9.71)	6.34 (5.53, 7.84)	5.39 (4.79, 5.64)	<0.001^abc^
Cr (μmmol/L)	75.00 (66.00, 92.00)	80.00 (71.50, 86.90)	66.50 (53.48, 78.00)	<0.01^bc^
Hb (g/L)	129.94 ± 21.28	141.33 ± 18.85	139.54 ± 14.76	<0.05^ab^
WBC (×10^9^/L)	5.79 ± 1.40	6.52 ± 2.01	5.49 ± 1.13	0.082
N (%)	64.63 ± 6.88	64.87 ± 7.48	57.24 ± 7.05	<0.001^bc^
L (%)	24.53 ± 6.79	25.70 ± 7.18	33.30 ± 6.60	<0.001^bc^

^*a*^ AF: atrial fibrillation; ALT: alanine aminotransferase; AST: aspartate transaminase; BMI: body mass index; CAD: coronary artery disease; Cr, creatinine; Hb: hemoglobin; HDL: high-density lipoprotein; L (%): lymphocyte ratio; LDH: lactate dehydrogenase; LDL: low-density lipoprotein; N (%): neutrophil ratio; NT-proBNP: N-terminal brain natriuretic peptide precursor; NYHA: New York Heart Association; RDW: red blood cell distribution width; STB: blood total bilirubin; TC: total cholesterol; TG: triglyceride; WBC: white blood cell. a is the statistical difference between HF-DS and HF-NDS, b is the statistical difference between HF-DS and HC, and c is the statistical difference between HF-NDS and HC.

^*b*^ –, not tested.

### Alteration of gut microbiota composition in HF-DS

We first conducted 16S rRNA sequencing for the collected fecal samples, and a total of 4,962 ASV representative sequences were obtained and annotated. Taxonomic analysis (Fig. S1A) demonstrated that at the phylum level, the top five bacteria in the three groups were *Bacteroidota*, *Firmicutes*, *Proteobacteria*, *Fusobacteriota*, and *Actinobacteriota*. In addition, based on the ternary phase diagram, we discovered that the relative level of *Fusobacteriota* was higher in the HF-DS, and the relative level of *Bacteroidota* was higher in the HF-NDS (Fig. S1B). Subsequently, we assessed the species abundance, diversity, and structural differences. The α-diversity index including Chao, Shannon, and Simpson revealed no statistical differences between the HC, HF-NDS, and HF-DS groups (Fig. S2A through C), indicating that there were no significant differences in microbiota abundance and diversity. Furthermore, we analyzed inter-sample differences (β analysis) by the unweighted UniFrac algorithm (Fig. S2D and E). The results showed significant heterogeneity in colony structure between the three groups (Anosim: stress = 0.133, *R* = 0.078, *P* = 0.001; Adonis: *R* = 0.043, *P* = 0.002).

**Fig 1:**
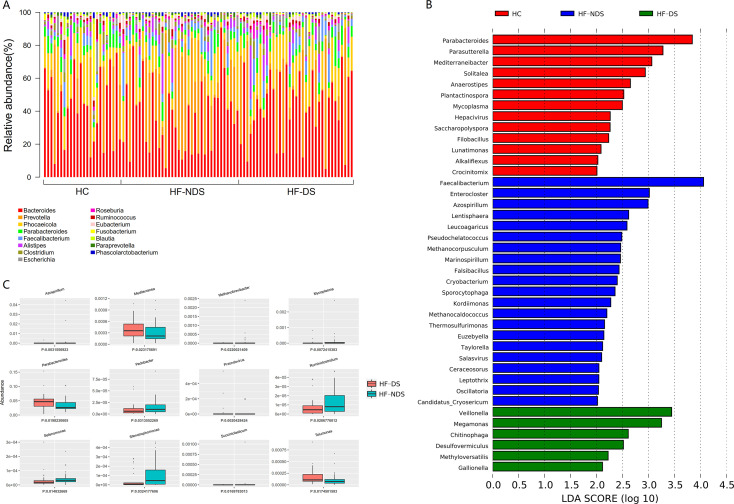
Determination of microbiota biomarkers specific for each HF stage. (**A**) Overview of the relative abundance of microbiota by genus level of the HC, HF-NDS, and HF-DS groups. (**B**) Gut microbiota that best characterizes HC, HF-NDS, and HF-DS groups was determined using LEfSe on genus-level abundance tables (*P* < 0.05, *q* < 0.1, LDA > 2.0). (**C**) The top 12 differential microbiota at the genus level of the HF-DS and HF-NDS patients (*P* < 0.05).

To further explore the taxonomic features and composition differences of gut microbiota associated with HF comorbid depression, the raw macrogenome data were checked by quality control, filtered, and assembled to construct abundance profiles at the corresponding taxonomic levels. Sequence analysis revealed that important genus-level features mainly include *Bacteroides*, *Prevotella*, *Phocaeicola*, *Parabacteroides*, *Faecalibacterium*, and *Alistipes* (Fig. 1A). In LEfSe analysis (*P* < 0.05, *q* < 0.1, LDA > 2.0), we noted different stages of pattern characteristics including *Veillonella, Megamonas, Megamonas*, and *Desulfovermiculus* (HF-DS); *Azospirillum*, *Prevotella,* and *Enterocloster* (HF-NDS); *Dialister* and *Anaerostipe* (HC) (Fig. 1B). In addition, *Mediterranea*, *Tolumona*, and *Parabacteroides* were more enriched in HF-DS than HF-NDS, while *Pedobacter*, *Azospirillum*, and *Ruminiclostridium* were further diminished (Fig. 1C).

**Fig 2:**
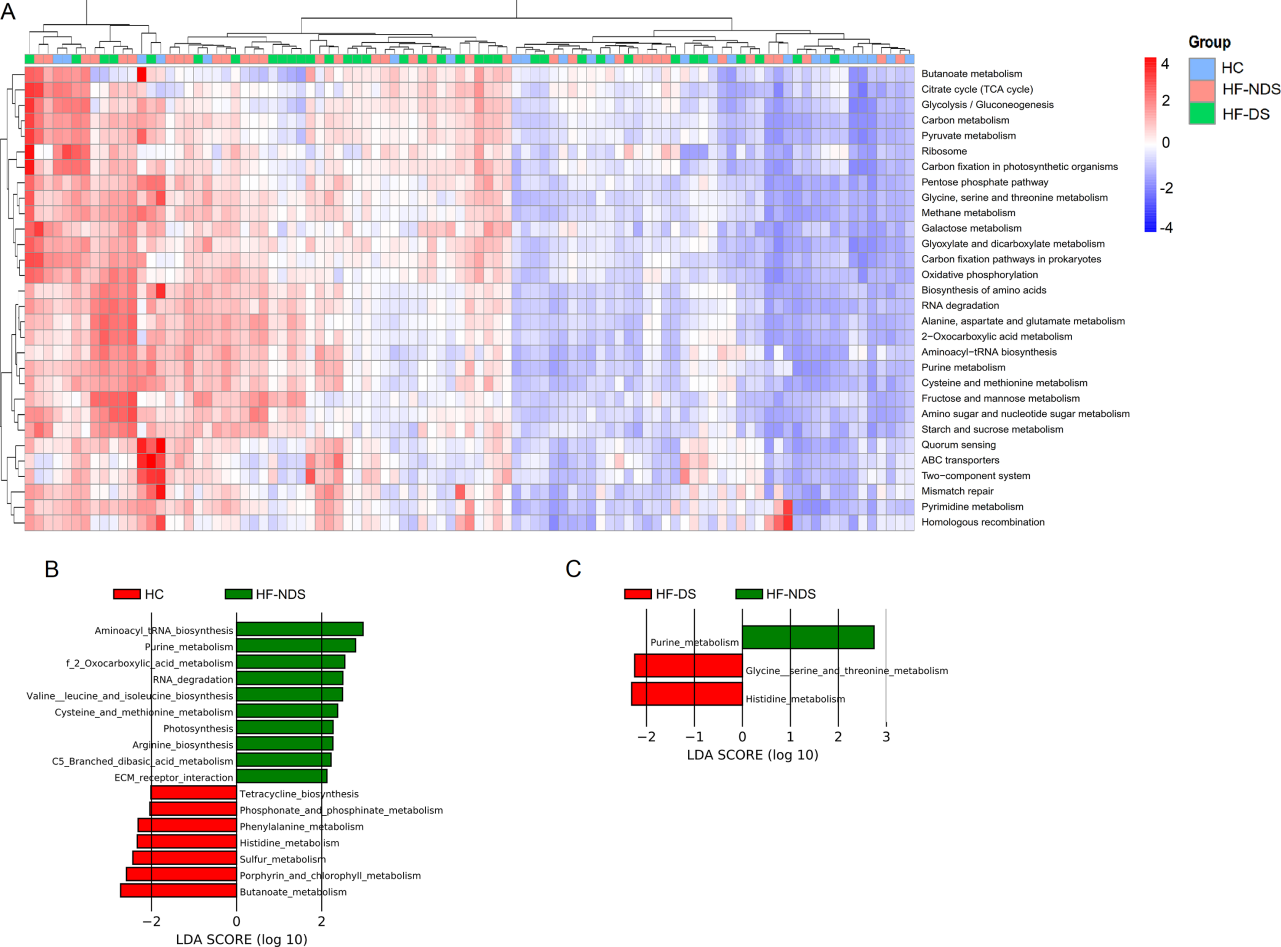
Functional pathway alterations involved in gut microbiota. (**A**) Overview of the key functional pathways enriched in HC, HF-NDS, and HF-DS groups. (**B**) Using LEfSe to identify key different pathways in the HF-NDS and HC groups (*P* < 0.05, *q* < 0.1, LDA > 2.0). (**C**) Using LEfSe to determine the key different pathways in the HF-DS and HF-NDS groups (*P* < 0.05, *q* < 0.1, LDA > 2.0).

We next mapped the bacterial gene catalog of macrogenome to KEGG database for examining the functional changes in the gut microbiota composition differences. LEfSe analysis (*P* < 0.05, *q* < 0.1, LDA > 2.0) showed (Fig. 2A through C) that the functional pathways involved in the dominant microbiota in HF-NDS when compared to HC were mainly cysteine and methionine metabolism, ECM receptor interaction, leucine and isoleucine biosynthesis, etc.; when compared to HF-NDS, the functional pathways involved in the dominant microbiota in HF-DS were mainly glycine, serine and threonine metabolism, and histidine metabolism. Thus, the abundance of relevant genes responsible for amino acid metabolism was further altered in HF-DS patients, indicating that abnormality in microbial-derived amino acid metabolism may be implicated in the development of depressive symptoms in HF patients.

### Alteration of gut metabolites in HF-DS

**Fig 3:**
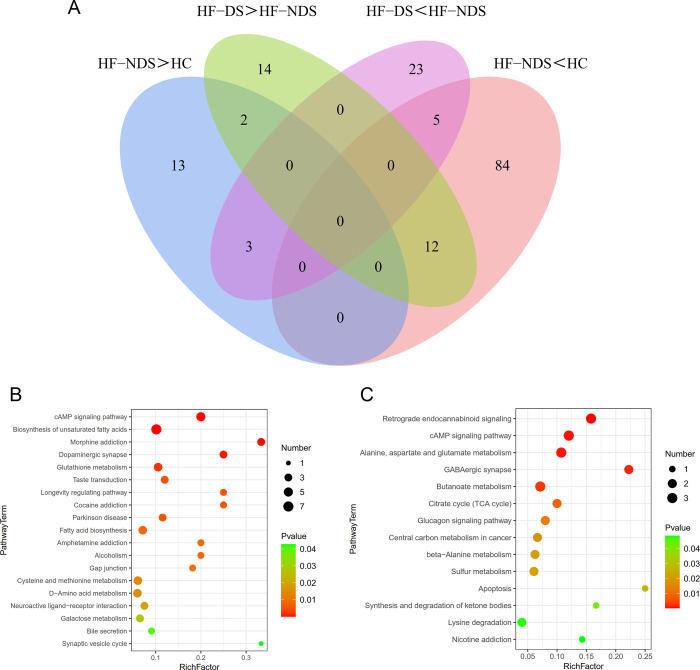
Functional pathway alterations involved in gut metabolic profiles. (**A**) Venn diagram showing the intersection of differential metabolites between HC, HF-NDS, and HF-DS groups (VIP > 1, *P* < 0.05). (**B**) By KEGG to determine the major differential pathways in the HF-NDS and HC groups (*P* < 0.05). (**C**) By KEGG to identify the key differential pathways in the HF-DS and HF-NDS groups (*P* < 0.05).

A combination of multidimensional and unidimensional analyses was used to screen for differential metabolites between groups (VIP value > 1, *P* < 0.05). A total of 119 differential metabolites were discovered in the HF-NDS vs HC (Fig. S3A through C), 115 differential metabolites were discovered in the HF-DS vs HC (Fig. S3D through F), and 59 differential metabolites were discovered in the HF-DS vs HF-NDS (Fig. S3D through F). Besides, anti-inflammatory mediators, including abietic acid, quinic acid, linoleic acid, and arbutin, were significantly lower in HF-NDS and HF-DS than HC; second, neurotransmitters, including catechin, caffeic acid, serotonin, tryptamine, beta-sitosterol, and phenylethylamine, were also impaired in synthesis. Several characteristic metabolites, including L-methionine, N-acetylaspartic acid, palmitic acid, and norvaline, were significantly decreased in HF-DS. It is noteworthy that N-acetyl-5-hydroxytryptamine and phthalic acid were synthesized more exuberantly in HF-DS than HF-NDS, while caffeic acid and quinic acid were further depleted. Collectively, these findings suggest that disturbances in intestinal metabolism are an important factor in depression in patients with HF (Fig. 3A). To verify whether the functional pathways involved in gut metabolites and microbiota are consistently altered, we performed pathway enrichment analysis on the KEGG IDs of metabolites to obtain pathway enrichment results. The enrichment analysis revealed that the differentially enriched pathways between HC and HF-NDS groups mainly included biosynthesis of unsaturated fatty acids, cAMP signaling pathway, glutathione metabolism, etc*.* (Fig. 3B); the differentially enriched pathways between the HF-NDS and HF-DS groups mainly included cAMP signaling pathway, GABAergic synapse, butanoate metabolism, etc*.* (Fig. 3C). Thus, anti-inflammatory and neurotransmitter-related gut metabolites and their pathways are linked to depressive symptoms in HF patients.

### Combined analysis of macrogenome and metabolomic

**Fig 4:**
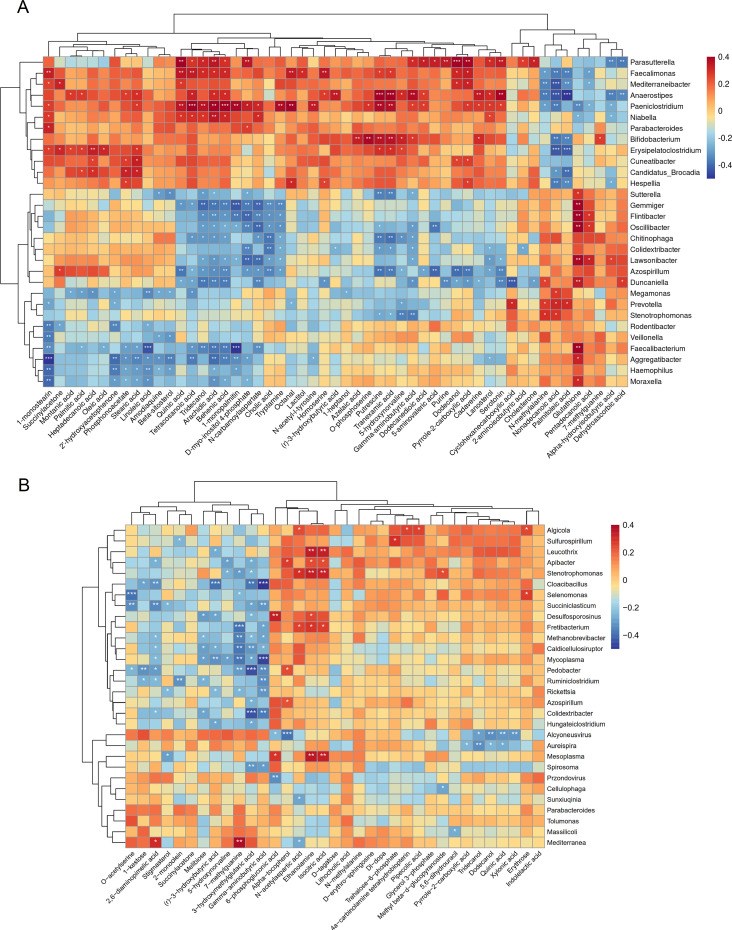
Potentially mechanistic associations of gut microbiota with metabolites. (**A**) Spearman’s correlation analysis of the top 30 microbiota and differential metabolites in the HF-NDS and HC groups. (**B**) Spearman’s correlation analysis of the top 30 microbiota and differential metabolites in the HF-DS and HF-NDS groups (*P < 0.05, **P < 0.01, ***P < 0.001).

Our multi-omics data enable multifaceted identification of microbiota and metabolite signatures. Currently, positive/negative correlations between microbiota and metabolites may imply that species mediate the production/consumption of related metabolites or that metabolites promote/inhibit the growth of related species. To identify the above relationships, we conducted a large-scale association analysis of the top 30 genus-level differential microbiota with organic and aliphatic differential metabolites, respectively. Using the Spearman algorithm, we identified 336 and 113 significant associations (*P* < 0.05) in the HF-NDS vs HC (Fig. 4A) and HF-NDS vs HF-DS (Fig. 4B), separately. The correlation analysis of HF-NDS vs HC indicated that *Parasutterella*, *Anaerostipes*, and *Paeniclostridium* were positively linked to most organic and aliphatic differential metabolites, while *Azospirillum* and *Aggregatibacter* were predominantly negative. Notably, the decrease in *Anaerostipes* was significantly linked to an increase in long-chain fatty acids such as cholesterone and nonadecanoic acid and a decrease in amino acid products such as GABA and serotonin. The association analysis between HF-NDS and HF-DS indicated that *Ruminiclostridium*, *Cloacibacillus*, *Colidextribacter*, and *Pedobacter* were strongly linked to metabolites. *Cloacibacillus* and *Colidextribacter* were negatively correlated with GABA, while *Pedobacter* and *Cloacibacillus* were negatively linked to 3-hydroxymethylglutaric acid (a significant intermediate metabolite of lipids). Collectively, there is a complex host-microbiota-metabolites biological network in the process of HF combined with depression.

### Random forest probes for multi-omics signature markers

**Fig 5:**
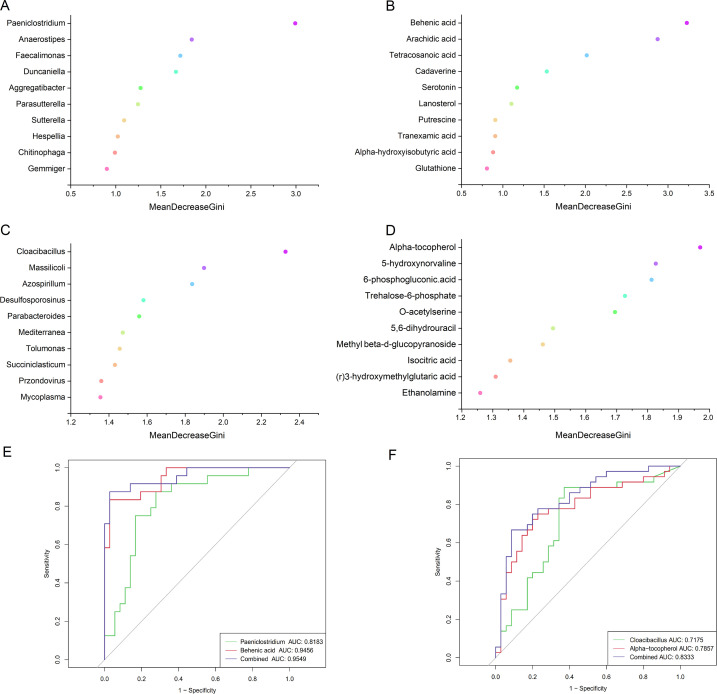
Metagenomic and metabolomic markers for determining HF-NDS (**A and B**) and HF-DS (**C and D**) patients were identified from random forests classifiers based on the combination of dual-omics markers. ROC curves depict trade-offs between true and false positive rates for detecting HF-NDS (**E**) and HF-DS (**F**) patients.

To evaluate the potential of gut genomic and metabolomic parameters as markers for the diagnosis of HF combined with depression, we constructed random forest regression models (Fig. 5A through D) to assess the differences in three groups of subjects by microbiota, metabolite, and a combination of both, respectively. By comparing the classification potential of the three models, we discovered that the combined model was higher than the single microbiota or metabolite model in differentiating HF-NDS from HC and HF-NDS from HF-DS, denoting that multi-omics feature had a higher diagnostic effect. In HF-NDS vs HC group (Fig. 5E), the area under the ROC curve for patients with HF-NDS diagnosed by the combined model was 0.955, and the ROC values for the microbiota model and metabolite model were 0.946 and 0.818, respectively. *Paeniclostridium* was a highly labeled microbiota, and the metabolite was behenic acid. The area under the ROC curve of the combined model for diagnosing HF-DS was 0.833, and the ROC values of the microbiota model and metabolite model were 0.718 and 0.786, respectively (Fig. 5F). Furthermore, *Cloacibacillus* and alpha-tocopherol were the major microbiota and metabolite features for determining HF-DS patients. Our outcomes show that diagnosing HF-DS patients by specific gut microbiota as well as metabolites is a viable strategy. Among them, *Cloacibacillus* and alpha-tocopherol had higher diagnostic efficacy.

## DISCUSSION

There is increasing evidence that alterations in gut microbial composition and function are associated with cardiovascular or psychiatric disease (6, 25, 26). Therefore, it is meaningful to investigate the taxonomic and functional characterization of the microbiota in HF patients who also have depressive symptoms. In this study, we identified specific gut microbiota and metabolite dysbiosis, as well as their functional alterations and interactions. These associations highlight the significance of host-microbiota-metabolites in the development of depressive symptoms in HF patients.

Among the altered composition of gut microbiota, we found two major patterns of species changes. One type of change (elevated or decreased) occurred across HF stages, while the other changed only during specific stages. Consistent with previous reports (27, 28), our study demonstrated that *Veillonella* was increased in both HF-DS and HF-NDS. Prior studies have reported that *Parabacteroides* are actively expressed in the GABA production pathway and can specifically cause SAMP6 mice to exhibit depression-like behavior (29). Similarly, we found that *Parabacteroides* was sequentially increased in HC, HF-NDS, and HF-DS and had a positive trend of correlation with GABA in the combined analysis. However, *Parasutterella* was decreased in both HF-DS and HF-NDS. As a vital part of the newly defined microbiota, *Parasutterella* was shown to be primarily involved in the maintenance of bile acid homeostasis and cholesterol metabolism (30), which may be linked to disorders of lipid metabolism in HF-DS. Furthermore, we discovered that *Pedobacter*, *Azospirillum*, and *Ruminiclostridium* were significantly lower in HF-DS than HF-NDS. HF and depression symptoms overlap, and the latter is frequently associated with decreased quality of life and poor clinical prognosis in HF patients, who may benefit from early diagnosis and intervention. We compared the taxonomic potential of differential microbiota by random forest analysis, and *Paeniclostridium* and *Cloacibacillus* were determined as the most important microbiota discriminating features for HF-NDS and HF-DS, respectively. These findings indicate that altered gut microbiota is linked to depressive symptoms in HF patients, and specific gut microbiota is an alternative diagnostic marker.

Metabolites are crucial mediators of communication between microbiota and host. Our study demonstrates that HC, HF-NDS, and HF-DS have significantly distinct gut metabolic profiles. Several anti-inflammatory mediators and neurotransmitters were differentially impaired in HF-NDS and HF-DS patients than HC, with disruptions in tryptophan metabolism being particularly common. Prior studies have revealed that gut microbiota can influence tryptophan metabolism and that its downstream metabolites can invade the blood-brain barrier to enter the central nervous system (CNS) (31, 32) and play an important role in neuronal function, immunity, aging, and other physiological processes (33). We found an impaired synthesis of serotonin, tryptamine, and phenylethylamine in HF-DS and HF-NDS than HC, although we did not find a consistent signal of change in the above products between HF-NDS and HF-DS. However, anti-inflammatory mediators such as alpha-tocopherol and quinic acid were further decreased in HF-DS. Previous studies have found that the activation of inflammatory factors elevates blood-brain barrier permeability and cascades to amplify the CNS damage of abnormal tryptophan metabolism (34, 35). Therefore, we attempt to speculate that the level of intestinal inflammation and abnormal tryptophan metabolism in HF patients plays a synergistic role in the generation of depressive symptoms.

Furthermore, we tentatively identified the relationship between host gut microbiota and metabolites. Spearman’s analysis indicated that a reduction in *Anaerostipes* was significantly linked to impaired synthesis of neurotransmitters linked to GABA and serotonin. A current study demonstrates that *Anaerostipes*, *Anaerostipes* sp., and related synthases could be promoted by supplementation with a GABA-rich diet to activate butyrate and propionate production (36). Second, we observed that *Parasutterella* and *Anaerostipes* showed a close link to adipose differential metabolites. *Parasutterella* is usually highly expressed in depression patients (37) and is closely associated with the activation of fatty acid biosynthetic pathway (38). Previous studies suggested that some strains of *Anaerostipes* could metabolize dietary inositol to SCFAs, such as propionate and butyrate (39). The latter is usually considered an important metabolite of the microbiota-gut-brain axis, and its reduced synthesis is closely linked to the development of depression (40). Taken together, the highly consistent abundance of disturbed gut microbiota and metabolites in HF-DS suggests that inter-crosstalk in the gut ecosystem may be a key factor in the development of depressive symptoms in HF patients.

The functional mechanisms of intestinal microecology in HF-DS are mostly unknown. Here we discovered comparable changes in the functional pathways involved in gut microbiota and metabolites. Amino acid-related metabolism and fatty acid-related metabolism may be important biological mechanisms implicated in the onset of depression in HF patients. Furthermore, GABAergic synapse, retrograde endocannabinoid signaling, and cAMP signaling pathway may also influence the etiological mechanisms of HF-DS. The ω−3 polyunsaturated fatty acids are precursors of endogenous cannabinoid biosynthesis (41), which enhances neurotransmission in depressed patients by inhibiting the inflammatory response in CNS and delaying the over-activation of the immune response (42). Previous study has found that butyrate acts as a ligand and activator of short-chain fatty acid receptors to aid intestinal motility, induce intestinal hormone peptides or mediate the release of serotonin from intestinal chromophores, and improve the resistance of the intestinal lining to inflammation and oxidative stress (43). Besides, ketone bodies converted after incomplete oxidation of butyric acid excite the brain, promote neuronal growth, release brain-derived neurotrophic factor (44), and inhibit histone deacetylase regulatory genes (45), which are employed clinically to improve stroke, prevent dementia, and treat depression (46). In addition to fatty acid metabolic pathways, intestinal microecology linked to amino acid metabolisms such as alanine, aspartate, glutamate metabolism, and histidine metabolism is significantly altered in HF-DS. Disturbances in the peripheral and central metabolism of amino acid neurotransmitters such as dopamine, glutamate, and GABA are particularly prominent in depression-related studies (47). A recent preclinical study indicated that the rapid antidepressant mechanism of ketamine was associated with a dose-dependent surge of glutamate in trisynaptic, causing upregulation of synaptic proteins downstream and increased BDNF activity (48). Although we have determined multiple key pathways implicated in HF-DS, the association mechanisms between the pathways should be further explored.

We believe future additional work is required to address several limitations of this study. First, although we were the first to describe the abnormality in gut microbiota in HF-DS patients, given the constraints of study centers and sample size, we need to independently verify the diagnostic performance of the combined marker groups for samples from different geographic regions to avoid overly optimistic diagnostic reports. Second, due to the scarcity of relevant studies to date, many of the details of the associations identified need to be fully and thoroughly corroborated by animal experiments. Finally, we were unable to control the subjects’ diets and medications and could not guarantee the collection of samples in a fasting state, which may have had an unfavorable impact on the reported intestinal microecology.

Conclusively, we suggest that gut microecological crosstalk is linked to HF-DS. Disturbances in microbial lipid and amino acid metabolism are critical features. Moreover, we developed a combined marker that can effectively differentiate between HC, HF-NDS, and HF-DS. Ultimately, these outcomes provide new directions to unravel the pathogenesis and diagnostic strategies of HF-DS patients.

## Data Availability

The microbiota data are available in the National Center for Biotechnology Information [NCBI (https://www.ncbi.nlm.nih.gov)], under the accession number PRJNA993098. The metabolites data are available in MetaboLights [MTBLS (https://www.ebi.ac.uk/metabolights)], under the accession number MTBLS8183. The STORMS checklist is available in the Mendeley Data Repository (https://data.mendeley.com/datasets/vkgzyz6997/1).

## SUPPLEMENTAL MATERIAL

Supplemental figures msystems.00515-23-s0001.docx Fig. S1 to S3.

ASM does not own the copyrights to Supplemental Material that may be linked to, or accessed through, an article. The authors have granted ASM a non-exclusive, world-wide license to publish the Supplemental Material files. Please contact the corresponding author directly for reuse.
