# Supplementary material for: Landscape of gut microbiota and metabolites and their interaction in comorbid heart failure and depressive symptoms: a random forest analysis study
Source: mSystems. 2023 Oct 26;8(6):e00515-23. doi: 10.1128/msystems.00515-23 (PMC10734515; doi:10.1128/msystems.00515-23)
Supplement: Supplemental figures — Fig. S1 to S3. [file msystems.00515-23-s0001.docx]

*Supplementary material*

**Landscape of gut microbiota and metabolites characteristics and their interaction in heart failure combined with depressive symptoms: a random forest analysis study**

Kai Huang^1#^, MD; Jiahao Duan^1#^, MD; Ruting Wang^1^, MD; Hangfeng Ying^1^, MD; Qinwen Feng^1^, MD; Bin Zhu^2^, MD; Chun Yang^3*^, MD, PhD; Ling Yang^1*^, MD, PhD

^1^Department of Cardiology, The Third Affiliated Hospital of Soochow University, Changzhou 213003, China

^2^ Department of Critical Care Medicine, The Third Affiliated Hospital of Soochow University, Changzhou 213003, China

^3^ Department of Anesthesiology and Perioperative Medicine, The First Affiliated Hospital of Nanjing Medical University, Nanjing 210029, China

^*^Correspondence to:

Ling Yang, MD, PhD, Department of Cardiology, The Third Affiliated Hospital of Soochow University, Changzhou 213003, China. Tel: +86 13616110633. Email: linda_yl@sina.com

Chun Yang, MD, PhD Department of Anesthesiology and Perioperative Medicine, The First Affiliated Hospital of Nanjing Medical University, Nanjing 210029, China. Tel: +86 18260062666. Email: chunyang@njmu.edu.cn


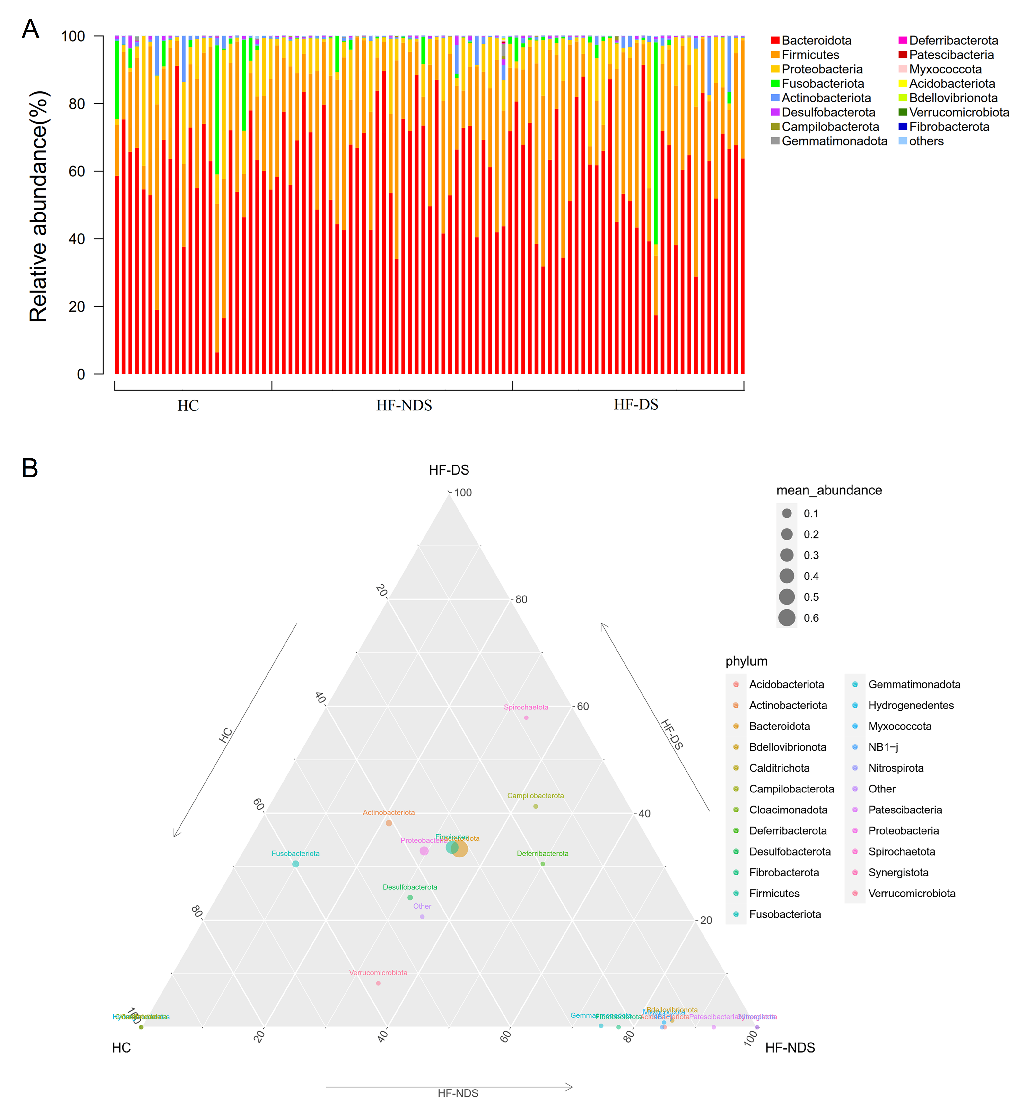


**Fig. S1** (A) Overview of the relative abundance of microbiota by phylum level of HC, HF-NDS, and HF-DS groups. (B) Ternary phase diagram showing the dominant phylum-level microbiota among HC, HF-NDS, and HF-DS groups.


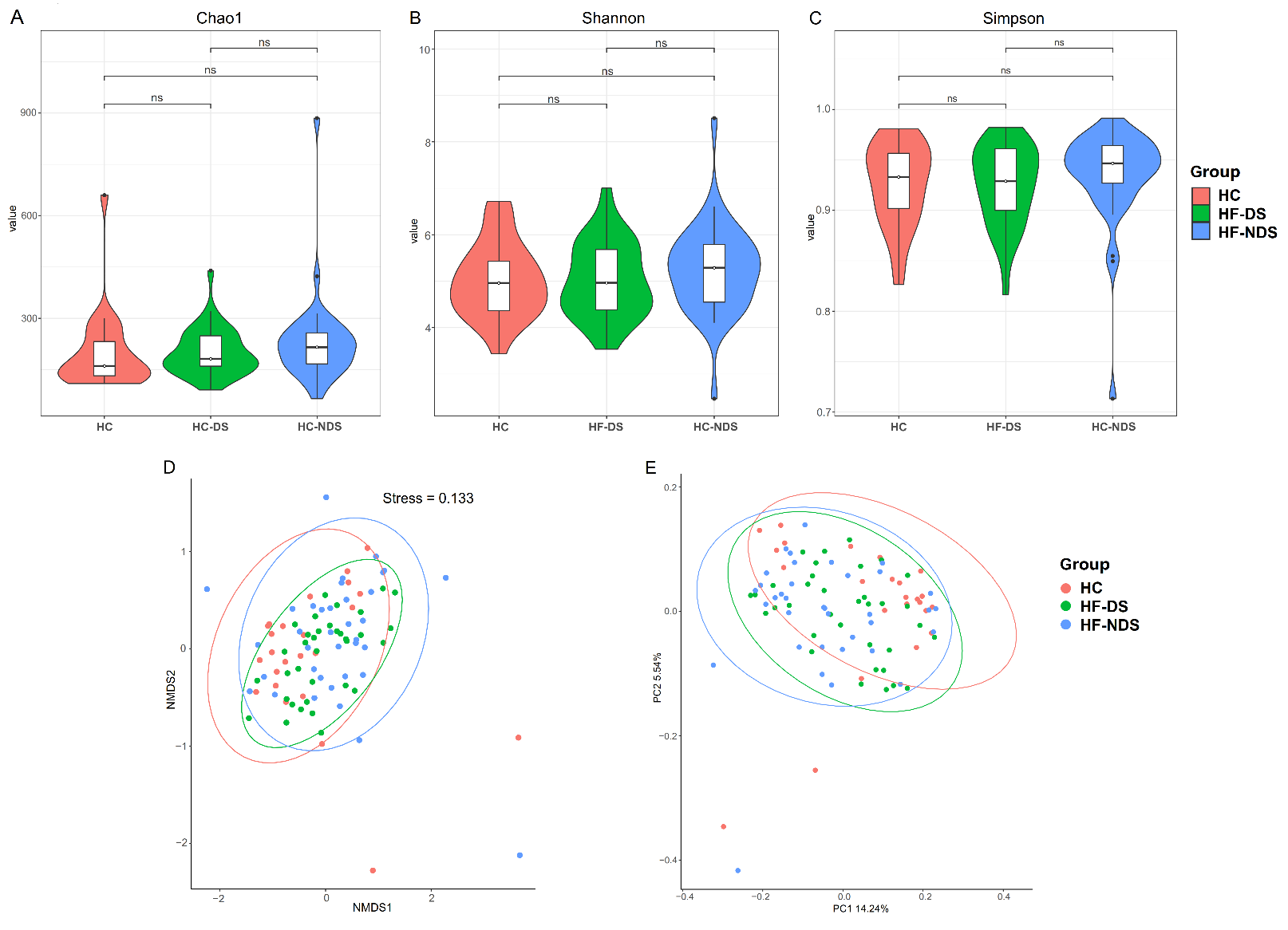


**Fig. S2 Severity-specific changes in microbiota diversities and taxonomic signatures.** (A-C) Differences in α diversity between HC, HF-NDS, and HF-DS groups. (D-E) Differences in β diversity between HC, HF-NDS, and HF-DS groups.


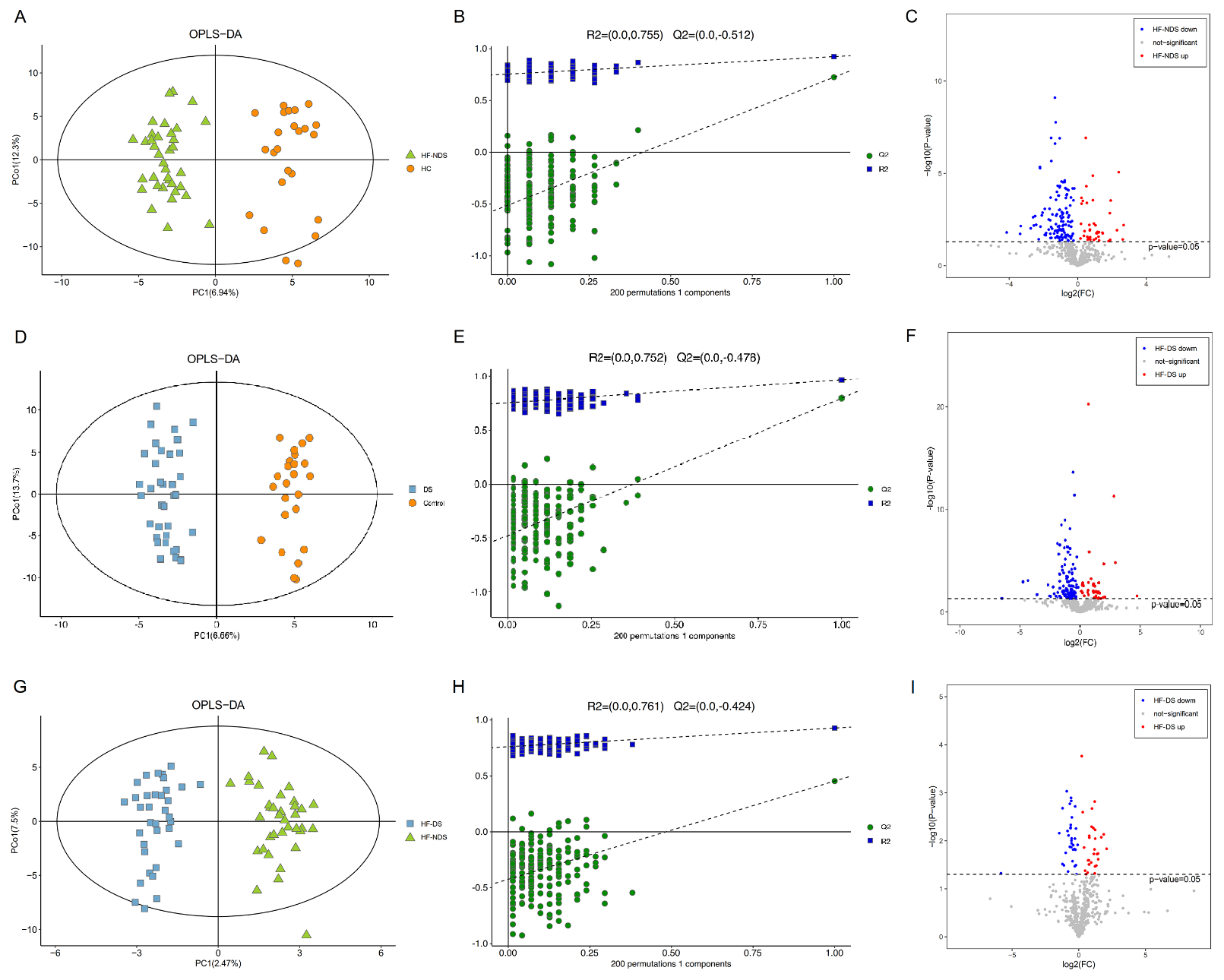


**Fig. S3 Multivariate and unidimensional statistical analysis of metabolic characters of stool samples acquired by GC-MS.** (A) Score plot of OPLS-DA between HF-NDS and HC. (B) Results of cross-validation between HF-NDS and HC. (C) Results of volcano plot between HF-NDS and HC. (D) Score plot of OPLS-DA between HF-DS and HC. (E) Results of cross-validation between HF-DS and HC. (F) Results of volcano plot between HF-DS and HC. (G) Score plot of OPLS-DA between HF-DS and HF-NDS. (H) Results of cross-validation between HF-DS and HF-NDS. (I) Results of volcano plot between HF-DS and HF-NDS.
